# Does tight glycemic control positively impact on patient mortality?

**DOI:** 10.1186/cc10783

**Published:** 2012-03-20

**Authors:** S Penning, AJ Le Compte, M Signal, P Massion, JC Preiser, GM Shaw, T Desaive, JG Chase

**Affiliations:** 1 Université de Liege, Belgium; 2 University of Canterbury, Christchurch, New Zealand; 3 CHU de Liège, Belgium; 4 Erasme University Hospital, Brussels, Belgium; 5 Christchurch Hospital, Christchurch, New Zealand

## Introduction

High and variable blood glucose (BG) levels have been associated with increased mortality. Tight glycemic control (TGC) aims at reducing BG levels to improve patient outcome and mortality. This research evaluates the impact of TGC on mortality.

## Methods

This study used glycemic data from 1,488 patients of two cohorts: Glucontrol (*n *= 704) and SPRINT (*n *= 784). TGC glycemic outcome is measured by cumulative time in the 4 to 7 mmol/l band (cTIB), defined daily for each patient. Each day, patients were divided into two groups: cTIB <70% and cTIB ≥70%. For each group, odds of living (OL = #lived/#died) was calculated.

## Results

**Figure 1:**
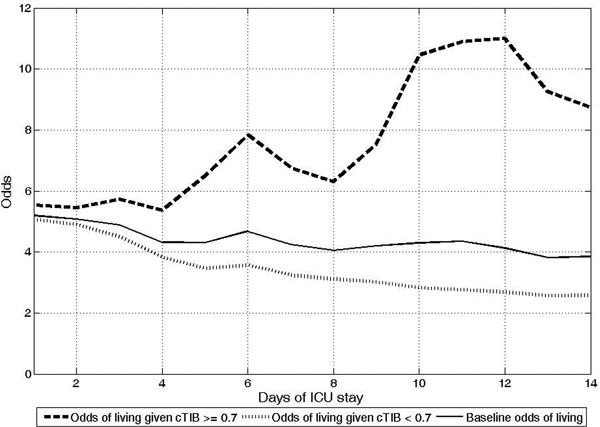
**Whole-cohort odds of living over ICU stay**.

OL for cTIB ≥70% patients tends to increase over time while OL for cTIB <70% patients decreases (Figure 1). On Day 1, OL for cTIB <70% patients and cTIB ≥70% patients are similar (OL = 5.1 and OL = 5.5 respectively). The difference between the two groups increases over the ICU stay. On Day 10, OL = 2.8 and OL = 10.5 for cTIB <70% and cTIB ≥70% patients respectively. These results suggest that survival rate is higher when cTIB ≥70% and thus when BG levels are tightly controlled around normoglycemia. The longer patients' ICU stay, the lower survival rate they have when cTIB <70%.

## Conclusion

Results show that, irrespective of TGC protocols, high cTIB and thus normoglycemia are associated with higher odds of living. This suggests that TGC positively influences patient outcome.

